# Co-inherited mutations of Fas and caspase-10 in development of the autoimmune lymphoproliferative syndrome

**DOI:** 10.1186/1471-2172-8-28

**Published:** 2007-11-13

**Authors:** Elisa Cerutti, Maria F Campagnoli, Massimo Ferretti, Emanuela Garelli, Nicoletta Crescenzio, Angelo Rosolen, Annalisa Chiocchetti, Michael J Lenardo, Ugo Ramenghi, Umberto Dianzani

**Affiliations:** 1 Interdisciplinary Research Center of Autoimmune Diseases (IRCAD) and Department of Medical Science, "A. Avogadro" University of Eastern Piedmont, Novara, Italy; 2 Department of Pediatrics, University of Turin, Turin, Italy; 3 Department of Pediatrics, University of Padua, Padua, Italy; 4 Molecular Development Section, National Institute of Allergy and Infectious Diseases, National Institutes of Health, Bethesda, MD, USA

## Abstract

**Background:**

Autoimmune lymphoproliferative syndrome (ALPS) is a rare inherited disorder characterized by defective function of Fas, autoimmune manifestations that predominantly involve blood cells, polyclonal accumulation of lymphocytes in the spleen and lymph nodes with lymphoadenomegaly and/or splenomegaly, and expansion of TCRαβ+ CD4/CD8 double-negative (DN) T cells in the peripheral blood. Most frequently, it is due to Fas gene mutations, causing ALPS type Ia (ALPS-Ia). However, other mutations, namely of the FasL gene (ALPS-Ib) and the caspase-10 gene (ALPS-II) are occasionally detected, whereas some patients do not present any known mutations (ALPS-III). Recently, mutations of the NRAS gene have been suggested to cause ALPS-IV.

**Results:**

This work reports two patients that are combined heterozygous for single nucleotide substitutions in the Fas and caspase-10 genes. The first patient carried a splice site defect suppressing allele expression in the Fas gene and the P501L substitution in caspase-10. The second had a mutation causing a premature stop codon (Q47X) in the Fas gene and the Y446C substitution in caspase-10. Fas expression was reduced and caspase-10 activity was decreased in both patients. In both patients, the mutations were inherited from distinct healthy parents.

**Conclusion:**

These data strongly suggest that co-transmission of these mutation was responsible for ALPS.

## Background

Autoimmune lymphoproliferative syndrome (ALPS) is a rare inherited disorder characterized by autoimmune manifestations that predominantly involve blood cells, polyclonal accumulation of lymphocytes in the spleen and lymph nodes with lymphoadenomegaly and/or splenomegaly, expansion of TCRαβ+ CD4/CD8 double-negative (DN) T cells in the peripheral blood and defective in vitro apoptosis of mature lymphocytes induced by the Fas death receptor [1-4]. Individuals with ALPS also have an elevated incidence of several types of lymphoma [5].

Fas belongs to the Tumor Necrosis Factor Receptor (TNFR) superfamily and induces cell death upon triggering by FasL [6,7]. It is highly expressed by activated effector lymphocytes in the immune response and switches it off by limiting clonal expansion of lymphocytes and favoring peripheral tolerance. Fas signaling starts from aggregation of Fas, the adaptor molecule FADD (Fas-associated death domain protein), and caspase-8 forming the Death Inducing Signaling Complex (DISC) which triggers caspase-8 activation and induces cell apoptosis through two partly interconnected pathways; the extrinsic pathway involves caspase-8-mediated direct activation of the cascade, whereas the intrinsic pathway proceeds through mitochondrial release of cytochrome c and activation of caspase-9. Both pathways converge in the activation of effector caspases, such as caspase-3, -6 and -7. In humans, but not in mice, the extrinsic pathway also involves caspase-10, that is recruited into the DISC and cooperates with caspase-8 in activation of the caspase cascade [8-10].

ALPS is generally due to deleterious mutations of the Fas gene (TNFRSF6) and is classified as ALPS type-Ia (ALPS-Ia) [11,12]. Other mutations, namely of the FasL gene in ALPS-Ib [13-15], and the caspase-10 gene (CASP10) in ALPS-II [16,17], are occasionally detected, whereas some patients do not present any known mutations (ALPS III)[1-3,18-20]. Recently, mutations of the NRAS gene have been suggested to cause a further type of ALPS (ALPS-IV) [21]. ALPS does not behave as a classical monogenic disease. Most ALPS type-Ia patients are heterozygous for the Fas mutation, but the parent carrying the mutation is generally healthy. Other complementary factors may thus be required in function of the severity of the mutation [22]. One possibility is that mild Fas mutations only induces ALPS when cooperate with mutations of other genes impairing function of the Fas system itself or other systems involved in similar functions. In line with this possibility, we have described osteopontin and perforin gene variations that predispose to ALPS [23,24]. The osteopontin gene variation correlated with production of increased amounts of this cytokine, which is involved in inflammation and also inhibits activation-induced cell death. The perforin gene variations were associated with decreased function of cytotoxic cells, which may switch off the immune response by fratricide of effector lymphocytes.

This work describes two unrelated patients that are double heterozygous for mutations of the Fas and the caspase-10 gene. Since the two mutations were inherited from distinct healthy parents, their co-transmission probably resulted in ALPS.

## Results

### Analysis of TNFRSF6 and CASP10

Pt.1 showed a heterozygous nucleotide substitution in TNFRSF6 (c334 -2a>g, [Genbank NM_000043.3]) located in the splicing-acceptor site in the third intron and determining the IVS3-2a>g splice site defect. The mutation results in skipping of exon 4, coding for an extracellular cysteine-rich domain, frameshift and premature termination after 38 codons. The mutated allele produces no protein. This mutation had already been described in a homozygous ALPS patient, whose heterozygous parents were healthy [25,26].

Sequencing of CASP10 detected a C>T substitution at nt1502 in exon 10 [Genbank NM_032977.2] resulting in a proline to leucine change (P501L) in the small catalytic subunit of caspase. The mutation, not previously described, was not detected in 80 healthy donors nor in 40 other ALPS patients. Family analysis showed that the CASP10 mutation was inherited from the apparently healthy mother, who did not carry the TNFRSF6 mutation. This was presumably inherited from the father, who was not available for analysis, or was a de novo mutation.

Pt.2 carried a heterozygous C>T substitution at nt139 in TNFRSF6. It was located in exon 2 coding for an extracellular domain and created a premature stop codon (Q47X). The mutation was predicted to cause haploinsufficiency due to nonsense mediated decay of the aberrant mRNA; alternatively, a truncated soluble Fas fragment might be produced. Sequencing of CASP10 detected a heterozygous nucleotide substitution (1337A>G) in exon 9 causing the Y446C amino acid change in the predicted protease domain of the small subunit. This variation has been previously associated to ALPS, but has been reported also in the healty Caucasian population with allelic frequency ranging from 1.6 to 2% [17,20]. Family analysis showed that the Fas mutation was inherited from the apparently healthy mother; the CASP10 variation was possibly inherited from the father, who was not available for the study.

**Figure 1:**
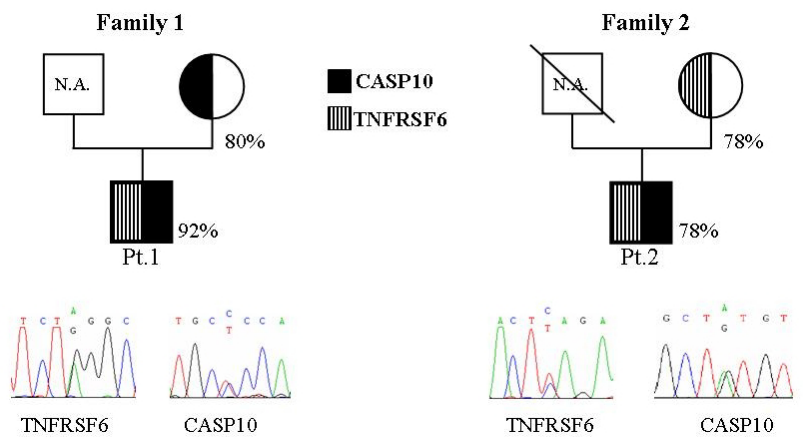
**Pedigrees of Family 1 and Family 2**. Inheritance of the CASP10 and TNFRSF6 mutations and electropherograms of the sequences performed on the genomic DNA of Pt.1 and Pt.2. Circles represent females; squares, males; subjects carrying a CASP10 mutation are marked in black, those with a TNFRSF6 are marked with striped lines. Numbers indicate the cell survival upon Fas triggering by mAb in T cell lines generated form each subject; Fas function was defective in Pt.1 and borderline in the other subjects (normal values of cell survival: median 60%, 95^th ^percentile 82%)

The pedigrees of these families are shown in the Figure 1.

### Functional analysis of the mutations

Analysis of Fas function in PHA-activated T cells from the patients and their mothers showed that it was defective in Pt.1 and borderline in Pt.2 since cell survival upon triggering of Fas was 92% in the former and 78% in the latter (normal values: median 60%, 95^th ^percentile 82%). Moreover, Fas function was borderline in both mothers since cell survival was 80% in the Pt.1's mother and 78% in the Pt.2 mother (Fig. 1).

**Figure 2:**
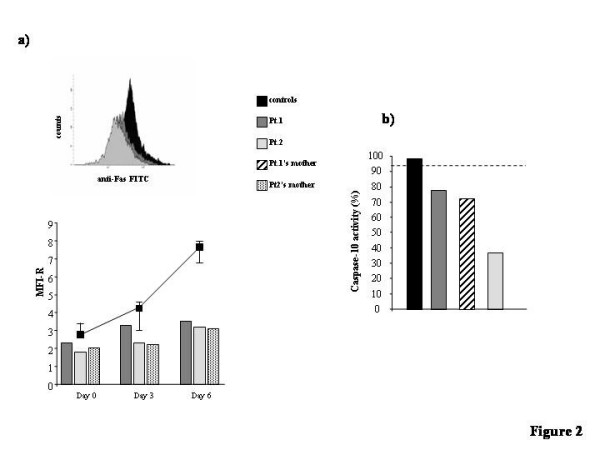
**Fas expression and caspase-10 activity in subjects carrying the TNFRSF6 and CASP10 mutation**. a) Fas expression was evaluated in T cell lines obtained by activating PBMC with PHA (1 μg/ml) and cultured for 6 days in RPMI 1640 +10% FCS+rIL-2 (2 U/mL). Before activation (day 0), and at day 3 and day 6 of culture, cells were stained with a FITC-conjugated anti-Fas mAb and analyzed with a cytofluorimeter. The *upper panel *shows the cytofluorimetric staining of cells from Pt.1, Pt.2, and a control donor after 6 days of culture. The *lower panel *shows the MFI ratio calculated for each subject at different times of culture. Control data are the medians ± interquartile ranges (25–75% range) from 5 control donors; their 5^th ^percentile value at day 6 was MFI-R = 6.48. b) Caspase-10 activity was evaluated in PHA-activated T cells cultured for 12 days (see Methods) in RPMI 1640 +10% FCS+rIL-2 (10 U/mL) and then treated or not with an anti-Fas mAb for 3 hours. Results are expressed as relative caspase activity % calculated as follows: (result displayed by each subject/mean of the results displayed by the 2 controls run in the same experiment) × 100; 100% indicates the mean of the results obtained with the 2 control donors run in parallel with the patient samples in each experiment; the dotted horizontal lines indicate the 5^th ^percentile of the activity displayed by all normal controls. The color code is the same in all panels.

To asses whether the IVS3-2a>g and Q47X TNFRSF6 mutations affected Fas expression, activated T cells from Pt.1 and Pt.2 were stained by direct immunofluorescence with an anti-Fas mAb and analyzed by flow cytometry. Results showed that both patients displayed decreased Fas expression (Fig. 2a).

To assess whether the P501L and Y446C caspase-10 amino acid substitutions affected the enzyme function, we evaluated caspase-10 activity induced by triggering of Fas with anti-Fas mAb in activated T cells from available mutated subjects, i.e. Pt.1, his mother, and Pt.2. All subjects showed a caspase-10 activity that was lower than that of the controls (Fig. 2b). These data suggest that both substitution decrease caspase-10 activity. Data on Y446C are in line with those previously reported [17] showing that the cloned Y446C-caspase-10 is less effective than the wild type form in restoring Fas-mediated apoptosis in cells lacking endogenous caspases-8 and -10, but does not exert dominant negative activity on the wild type.

**Figure 3:**
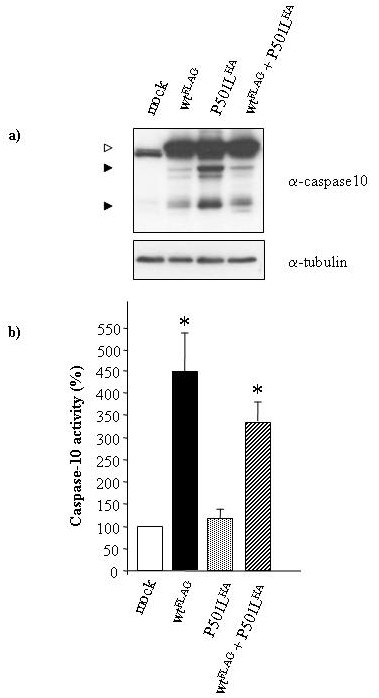
**Caspase-10 activity in 293T cells transfected with P501L-caspase-10**. Analysis of 293T cells transiently transfected with the mock, WT^FLAG^, P501L^HA^, or P501L^HA^+WT^FLAG ^plasmids, as indicated in the panel. a) Western blot analysis of cell transfectant lysates performed with anti-caspase-10 antibody; expression of the transfected molecules was confirmed using anti-HA and -FLAG antibodies (data not shown). The white arrow shows the pro-caspase-10; black arrows indicate the cleaved forms. b) Fluorimetric enzyme assay of caspase-10 activity evaluated in the cell transfectant lysates 24 h after transfection; data are relative to those displayed by mock-transfected cells (indicated as 100%) and are the means ± SE of data from 6 independent experiments. The asterisks mark the data significantly different from those obtained with P501L^HA^-transfected cells (p < 0.01, Mann Whitney test).

To further assess the activity of the novel P501L-caspase-10, the cDNAs coding for it or the wild-type protein (isoform d) were cloned into the pcDNA3.1 Myc-His vector, fused to HA- or FLAG-tag sequences respectively (P501L^HA ^and WT^FLAG ^plasmids). Both were transiently transfected into 293T cells, expressing minimal levels of endogenous caspase-10. Moreover, 293T cells were cotrasfected with the P501L^HA ^and WT^FLAG ^plasmids to determine whether the mutated form exterted a dominant negative activity on the wild type form. Western blot analysis showed that both constructs were expressed at comparable levels in all trasfectants and both proteins were spontaneously cleaved, the mutated form even more efficiently than the wild type form (Fig. 3). However, analysis of the caspase-10 enzyme activity on the lysates by a fluorimetric assay showed that the P501L-caspase-10 displayed about 50% of the activity displayed by the wild type. Cells cotransfected with the P501L^HA ^and WT^FLAG ^plasmids showed levels of caspase-10 activity intermediate between those of cells transfected with each plasmid alone, which indicates that the mutated form does not exert a dominant negative activity on the wild type.

## Discussion

This work reports that mutations of TNFRSF6, characterizing ALPS type Ia, and CASP10, characterizing ALPS type II, can cooperate in the development of ALPS.

Fas mutations are the most frequent in ALPS. Usually heterozygous, their penetrance depends on their effect on Fas function. Mutations hitting the intracellular death domain of Fas, involved in recruitment of FADD and caspase-8 and initiating the death signal, are often the most severe. They exert a dominant-negative effect and display high penetrance. By contrast, mutations hitting the extracellular portion or causing haploinsufficiency have weaker penetrance [22,27].

Both our patients carried mutations predicted to cause haploinsufficiency. In line with this prediction, T cells from both Pt.1 and Pt.2 expressed low levels of Fas. Penetrance of these mutations is presumably weak. This is suggested for the nonsense Q47X mutation by the lack of any sign of ALPS in the Pt.2's carrier mother. By contrast, the absence of Pt.1's father meant that clinical effect of IVS3-2a>g splice site defect could not be determined by a pedigree analysis, since it might have been a de novo mutation. However, this mutation has been previously described in a different family, where its heterozigosity was not sufficient to cause ALPS [26].

Only two CASP10 mutations have so far been unequivocally involved in ALPS. They are the missense mutations causing the L285F and I406L amino acid substitutions detected in 1 and 3 heterozygous patients respectively. Both mutations decreased caspase-10 activity and exerted a dominant negative effect on the wild type protein, but neither was sufficient to induce the overt disease, since several mutated familial components were healthy, and some displayed serum autoantibodies only [16,17].

Both CASP10 mutations carried by our patients are mild heterozygous missense mutations decreasing caspase-10 activity without exerting a dominant negative effect on the wild type protein. It is therefore intriguing that these patients also displayed mild Fas mutations that were presumably required to worsen the apoptotic defect and cause ALPS development. Pt.2 carried the Y446C mutation, previously reported in one heterozygous patient displaying a mild form of ALPS [17]. This mutation decreased caspase-10 function, without inducing a dominant negative effect on the wild type protein. The mutation is not sufficient to induce ALPS since it is also detected in 1–2% of the healthy Caucasian population [17,20]. Pt.1 carried the novel P501L mutation, located, like Y466C, in the small subunit of caspase-10. P501L, too, decreased caspase-10 function without inducing a dominant negative effect on the wild type protein. Decreased activity was clearly detected in lymphocytes from both this patient, who also carried the Fas mutation, and his mother, who only carried the CASP10 mutation. Moreover, 293T cells transfected with the mutated form displayed about 50% of the enzyme activity displayed by the wild type. Lack of negative dominance was shown when cotransfection with both the mutated and the wild type forms produced an additive and not an antagonistic effect on caspase-10 enzyme activity. It is noteworthy that western blot analysis of transfected cells showed that P501L did not affect cleavage of caspase-10, which is often interpreted as an evidence of activation. However, the decreased activity detected by the in vitro caspase-10 enzyme assay supports the model proposed by Boatright et al. that cleavage is neither sufficient or necessary for activation of initiator caspases, that mainly depends on dimerization [28].

The effect of the interaction between these Fas and caspase-10 mutations seems opposite to those reported for interactions between Fas mutations and caspase-10 missense variation V410I [16,17]. This variation was initially posited as a cause of ALPS since it had been detected in one homozygous patient. It has since been found as an "innocent" polymorphism carried by 3–5% of the Caucasian population [16,17,29]. An association analysis, indeed, suggested that it gave protection against severe ALPS in 63 families with ALPS-Ia caused by severe dominant mutations of Fas [17]. It is intriguing that the first ALPS patient homozygous for V410I harbored a heterozygous missense mutation in the tumor necrosis factor receptor-1 gene (TNFRSF1A), which is mutated in the TNF receptor-associated periodic fever syndrome (TRAPS) and may explain its clinical pattern [30]. This suggests that the caspase-10 variation also influenced the clinical phenotype due to the TNFRSF1A mutation.

## Conclusion

This work suggests that ALPS may sometimes be caused by the concurrent effect of mutations hitting different genes involved in Fas function and hence that it may be both a classic monogenic disease, as occurs in the presence of severe mutations hitting the intracellular portion of Fas, and the outcome of digenic or even oligogenic mutations affecting different steps of the Fas signalling pathway.

## Methods

### Patients

Patient 1 (Pt.1) was a 27-year-old Caucasian male. At the age of 23, he presented fever of unknown origin associated with mucositis, weight loss and nocturnal sweating. Laterocervical, axillary and inguinal lymphadenopathies with hepatomegaly and splenomegaly were clinically disclosed. Blood analyses showed reduced white blood cell and platelet counts, borderline hemoglobin levels, positive Coombs test and hypergammaglobulinemia, and expansion of DN T cells in the peripheral blood (6%). Histopathologic examination of left axillary lymph nodes showed reactive follicular hyperplasia with regular distribution of T- and B-dependent areas. Defective Fas-induced apoptosis of lymphocytes in cell death assays pointed to ALPS. Oral steroids improved the clinical picture, reduced lymph nodes and spleen size, and increased the platelet count and hemoglobin level.

Patient 2 (Pt.2) was a 12-year-old Caucasian male who displayed an ALPS phenotype at the age of 3, with laterocervical lymphadenopathy, mild hepatosplenomegaly, immune neutropenia and thrombocytopenia. Histopathologic examination of a submaxillary lymph node demonstrated follicular hyperplasia. DN T cells were 3.6% and Fas-induced lymphocyte apoptosis was borderline. Blood analyses also showed increased IgM and IgA levels and lupus anticoagulant. At the age of 4, he developed transient mild hemolytic anemia; hematological alterations spontaneously remitted at the age of 5, but laterocervical adenopathy increased to bulky masses. The left submaxillary and cervical lymph nodes began to compress the upper airways and were excised when he was ten years old; he is now in hematological remission, with persisting bulky laterocervical lymphadenopathies. The patient's father died of peritoneal carcinosis at 33. The paternal grandfather had died of gastric cancer.

### Sequence analysis

Genomic DNA was extracted from PBMCs by standard methods (Pure Gene DNA Isolation Kit, Gentra Biosystem Unc, Minneapolis, Minnesota) after informed consent. All coding exons and intron-exon boundaries of TNFRSF6 [Genbank AY450925.1] and CASP10 [Genbank NT_005403.14] were amplified by polymerase chain reaction (GeneAmp PCR System 9700, Applied Biosystems, Foster City, CA); primer sequences and annealing temperatures for each primers pair used for amplification were previously reported [20,31]. The PCR products were sequenced with the BigDyeTM Terminator Kit (Applied Biosystems,) on an ABI PRISM 3100 Genetic Analyzer (Applied Biosystems) using the same primers. The family segregation of mutations was ascertained by enzymatic digestion.

### Fas function assay

Fas-induced cell death was evaluated as previously reported on T cell lines obtained by activating PBMCs with PHA at days 0 (1 μg/ml) and 15 (0.1 μg/ml) and cultured in RPMI 1640 + 10% FCS + rIL-2 (2 U/ml) (Biogen, Geneva, Switzerland). Fas function was assessed 6 days after the second stimulation (day 21) [18,19]. Cells were incubated with control medium or anti-Fas mAb (CH11, IgM isotype) (1 μg/ml) (UBI, Lake Placid, NY) in the presence of rIL-2 (1 U/ml) to minimize spontaneous cell death. Cell survival was evaluated after 18 h by counting live cells in each well by the trypan blue exclusion test and by flow cytometry of cells excluding propidium iodide and unstained by annexin V-FITC; the two methods gave overlapping results. Assays were performed in duplicate. Cells from two normal donors were included in each experiment as positive controls. Results were expressed as specific cell survival %, calculated as follows: (total live cell count in the assay well/total live cell count in the control well) × 100. Fas function was defined as defective when cell survival was >82 % (the 95th percentile of data obtained from 200 normal controls).

### Immunophenotype analysis

Expression of surface molecules was evaluated by direct immunofluorescence and flow cytometry (FACScan. Becton Dickinson, San Jose, CA). The following mAb were used: anti-CD4 (Leu-3a), -CD8 (Leu-2a), -TCRαβ (Becton Dickinson), and -Fas (Immunotech, Marseilles, France). CD4 and CD8 DN TCRαβ-positive cells were detected by 2-color immunofluorescence with fluorescein isothiocyanate (FITC)-conjugated anti-TCRαβ mAb and phycoerythrin (PE)-conjugated anti-CD4 and anti-CD8 mAbs. Fas was detected by 2-color immunofluorescence on resting or activated T cells, using PE-conjugated anti- TCRαβ mAb and FITC-conjugated anti-Fas mAb (Chemicon, Temecula, CA). Nonspecific background fluorescence was established with the appropriate isotype-matched control mAb (Becton Dickinson)

### Caspase-10 activity

PBMCs were activated with PHA at days 0 (1 μg/ml) and 8 (0.1 μg/ml) and cultured in RPMI 1640 + 10% FCS + rIL-2 (10 U/ml). Four days after the second stimulation, 6 × 10^6 ^T cells were treated or not with anti Fas mAb (CH11, IgM isotype) (1 μg/ml) on ice for 30 min, then moved to 37°C for 3 h and centrifuged. At least 2 control samples, using T cells from different healthy donors, were always run in parallel. Caspase-10 activity of T-cells and transfected 293T was assessed in cell lysates using a fluorimetric assay (MBL, Watertown, MA).

### Caspase-10 cloning

cDNA coding for wild type caspase-10 was obtained as previously reported [16]. To obtain the cDNA of mutated caspase-10, we amplified the fragment containing the P501L mutation, from 1055 bp to 1569 bp [Genbank NM_032977.2] by RT-PCR. Briefly, total RNA extracted from patient's PBMC was reverse-transcribed with the ThermoScript RT-PCR system (Invitrogen, Milan, Italy) and amplified with primers C10 NcoI-fw and C10 XhoI-rev (Table 1). The fragment from ATG to 1055 bp was obtained from the wild type clone by PCR using a 5'-primer adding the HA TAG (C10HA-fw/C10 NcoI-rev). In order to discriminate between the wild type and the mutated form, we added the FLAG TAG to the wild type clone by PCR (C10FLAG-fw/NcoI-rev). Both cDNAs were then cloned into the pcDNA3.1 Myc-His vector (Invitrogen) and sequenced.

**Table 1:** Primers used for caspase-10 cloning

C10HA-*fw*	5'-ccgaattcatgtacccttatgatgtgccagattatgcctctaaatctcaaggtcaacattgg-3'
C10FLAG-*fw*	5'-ccgaattcatggactacaaggacgacgatgacaagaaatctcaaggtcaacattgg-3'
C10NcoI-fw	5'-gttctgtattctgacccatgggag-3'
C10NcoI-*rev*	5'-tccaaatctcccatgggtc-3'
C10XhoI-*rev*	5'-ccgctcgagtaatgaaagtgcatccag-3'

### Transient Transfection and Western Blotting

Human embryonic kidney 293T cells (ATCC #CRL-11268) were cultured in Dulbecco's modified essential medium (DMEM) supplemented with 10% FCS at 37°C. 3 × 10^6 ^cells were plated in 10 cm dishes and transfected with 24 μg of the empty vector, the WT^FLAG ^vector, the P501L^HA ^vector, or a mix of them by Lipofectamine 2000 kit (Invitrogen). After 24 h, adherent and floating cells were harvested in lysis buffer (MBL) for 30 min. Cell debris were removed by centrifugation and equal amounts of the cleared lysates were heated for 5 min at 95°C. Protein extracts were then separated by SDS-PAGE, transferred to Hybond-C extra membranes (Ge Healthcare, Piscataway, NJ, USA), blotted with anti-caspase-10 (1 μg ml^-1^) (MBL), anti-HA (1 μg ml^-1^) (Santa Cruz Biotechnology, Santa Cruz, CA, U.S.A.), anti-FLAG (1 μg ml^-1^) (Sigma, Milan, Italy), anti-tubulin (1 μg ml^-1^) (Sigma) and a peroxidase-conjugated anti-mouse or rabbit antibodies (Ge Healthcare), and revealed by chemiluminescence.

## Authors' contributions

UD, EC and AC drafted the manuscript, all authors contributed to the revision. EC, MFC, MF, EG, NC performed experiments. AR diagnosed one patient and contributed to the manuscript writing. LG was responsible for data collection and analysis. ML critically revised the paper and is responsible for important intellectual content. UD and UR were involved in the conception and design of the study. All authors edited and approved the written manuscript.
